# The prevalence of thyroid nodules in northwest China and its correlation with metabolic parameters and uric acid

**DOI:** 10.18632/oncotarget.14720

**Published:** 2017-01-18

**Authors:** Yao Liu, Ziwei Lin, Chunjun Sheng, Yikun Zhu, Yun Huang, Ni Zhong, Zhao Jia, Shen Qu

**Affiliations:** ^1^ Department of Endocrinology and Metabolism, Shanghai Tenth People’s Hospital, School of Medicine, Tongji University, Shanghai, China; ^2^ Department of Endocrinology and Metabolism, The Second Hospital of Shanxi Medical University, Shanxi, Taiyuan, China

**Keywords:** thyroid nodule, serum uric acid, metabolic disorders, prevalence, epidemiology

## Abstract

This study aimed to estimate the prevalence of thyroid nodules (TN) and investigate its correlation with metabolic parameters, especially uric acid (UA) in northwest Chinese population. We conducted a large cross-sectional survey with 67,781 residents (33,020 men, 34,761 women), aged from 18 to 86 years in Shanxi, China, from January 2012 to December 2014. A thyroid ultrasound examination was performed with number and size of nodules being recorded. Metabolic parameters including body mass index (BMI), blood pressure (BP), triglycerides (TG), total cholesterol (TC), low-density lipoprotein cholesterol (LDL-c), high-density lipoprotein cholesterol (HDL-c), fasting glucose (FG), and uric acid (UA) were also examined. Our study revealed that approximately 30.7% of men and 39.9% of women in Northwest China had TN, about half of which were multi-nodularity and a quarter of their TN larger than 1 cm. The prevalence of TN increased with aging and increasing BMI, and metabolic disorders, which also related to the increased incident of multi-nodularity and larger TN. Serum UA appeared to be a protective factor for TN in men older than 30 years, but a risk factor in both men younger than 30 years and women older than 30 years. This phenomenon needs to be further investigated.

## INTRODUCTION

Thyroid nodule (TN) is a clinical common hyperplastic disease with 2-5% being malignant. In recent years, the prevalence of TN has increased significantly and is now reaching epidemic proportions in China. In 2002 an epidemiological survey in Northeast China found that the prevalence of TN was about 8% [1], which reached 25% in 2013 [2]. The rapid increase in TN might be partly due to the improved screening procedures. As shown in an epidemiological survey, the prevalence rate of TN is about 5% in women and 1% in men with palpation, and 19% to 68% with high resolution ultrasound respectively [3].

It has long been recognized that TN is associated with iodine deficiency or excess, genetic, immune, endocrine disruptors, or exposure to radiation and drug [4, 5]. Recent studies have found that the metabolic disorders have also played an important role in TN [6, 7]. A clinical study in a mild-to-moderate iodine-deficient area showed that the percentage of patients with TN was 50.4% in metabolic syndrome group, while the percentage was only 14.6% in control group. The study also found insulin resistance was a risk factor for TN.

Uric acid (UA) is the end product of purine metabolism in the human body. It is closely related to type 2 diabetes mellitus, coronary heart disease, and hypertension. It has been found to be one of the most important parts of metabolic syndrome [8–10]. However, the relationship between TN and UA was unknown.

To address this knowledge gap, we studied the ultrasound results of thyroid from a large sample of 67,781 residents attending a Health Examination Center, in Shanxi, China from 2012 to 2014. We further investigated the relationship between characteristics of TN and metabolic parameters, especially UA.

## RESULTS

### Metabolic characteristics in people with TN

The characteristics of study subjects are shown in Table 1 and Table 2. This study included 67,781 subjects (33,020 men, 34,761 women), aged from 18 to 86 years. The prevalence of TN was 35.4% in the study population, 30.7% in men and 39.9% in women. Among the subjects with TN, 49.9% were with multiple nodules (47.2% in men and 51.8% in women) and 23.1% were with nodules diameter larger than 1cm (21.2% in men and 24.5% in women). As shown in Table 1, measured variables were compared by TN occurrence, TN count, and TN size. Subjects with TN tended to have a worse metabolic status, and more likely to be older, fatter, with higher blood pressure (both SP and DP), worse serum lipid (higher TC, TG, LDL), and higher glucose. Metabolic status was even worse in subjects with multiple nodules compared to those with single nodule, and in subjects with larger nodules (≥1cm) compared to subjects with smaller nodules ( < 1cm). However, UA presented an opposite trend which was slightly decreased in subjects with multiple and larger nodules.

**Table 1:** General characteristics and levels of metabolic components in the thyroid nodule group, thyroid nodule count group and thyroid nodule size group

	Thyroid Nodule		Thyroid Nodule Count		Thyroid Nodule Size	
	TN Absent (*n*= 43771, 64.6%)	TN Present (*n*= 24010, 35.4%)	Single TN (*n*= 12039, 50.1%)	Multiple TN (*n*= 11971, 49.9%)	TN <1cm (*n*= 18466, 76.9%)	TN ≥1cm (*n*= 5544, 23.1%)
Age (years)	40.52±12.60	48.93±13.91*	47.63±13.29	50.40±14.32△	50.88±13.78	52.24±13.20^▲^
BMI (kg/m2)	24.48±3.58	25.03±3.42*	24.95±3.43	25.11±3.40△	25.43±3.48	25.87±3.26
SBP (mmHg)	124.31±17.87	129.26±19.70*	128.06±19.37	130.48±19.95△	131.29±20.87	132.50±18.97^▲^
DBP (mmHg)	76.99±12.01	79.06±12.25*	78.78±12.21	79.35±12.29△	79.98±12.32	80.83±11.66^▲^
TG(mmol/L)	1.55±1.11	1.63±1.08*	1.62±1.09	1.65±1.07	1.68±1.08	1.69±0.98
TC(mmol/L)	4.55±0.89	4.70±0.92*	4.67±0.91	4.73±0.93△	4.77±0.96	4.88±0.90^▲^
HDL(mmol/L)	1.34±0.35	1.33±0.34	1.33±0.34	1.34±0.35	1.33±0.36	1.33±0.36
LDL (mmol/L)	2.57±0.73	2.68±0.76*	2.66±0.76	2.69±0.77△	2.68±0.80	2.69±0.78
FG (mmol/L)	5.42±1.18	5.64±1.45*	5.58±1.36	5.70±1.53△	5.74±1.55	5.90±1.60^▲^
UA(umol/L)	309.41±86.53	300.32±82.75*	301.60±83.09	299.03±82.39△	304.50±89.63	300.50±85.56^▲^

*P < 0.01 *vs*. Thyroid Nodule Absent group. △*P* < 0.01 *vs*. Single Thyroid Nodule group. ^▲^*P* < 0.01 *vs*. Thyroid Nodule<1cm group.

**Table 2:** Comparison of the levels of metabolic components in male group and female group

				**Male**							
	**TN Absent** (***n*= 22891, 69.3%)**	**TN Present** (***n*= 10129, *n* = 30.7%)**		**Single TN** (***n*= 5344, 52.8%)**		**Multiple TN** (***n*= 4785, 47.2%)**		**TN<1cm** (***n*= 7986, 78.8%)**		**TN≥1cm** (***n*= 2143, 21.2%)**	
Age (years)	41.54±12.90	49.97±14.27*		48.69±13.72		51.40±14.72△		49.31±14.12		52.42±14.56^▲^	
BMI (kg/m2)	25.47±3.43	25.87±3.21*		25.80±3.26		25.96±3.17△		25.85±3.24		25.98±3.13	
SBP (mmHg)	128.62±17.31	132.94±18.78*		131.86±18.48		134.14±19.04△		132.65±18.66		134.02±19.20^▲^	
DBP (mmHg)	80.58±11.73	82.74±12.01*		82.39±11.89		83.13±12.13△		82.61±12.07		83.26±11.77^▲^	
TG(mmol/L)	1.84±1.25	1.88±1.21*		1.88±1.21		1.88±1.20		1.88±1.21		1.88±1.20	
TC(mmol/L)	4.60±0.88	4.66±0.88*		4.66±0.88		4.65±0.89		4.66±0.88		4.64±0.91	
HDL(mmol/L)	1.22±0.31	1.19±0.29*		1.20±0.30		1.19±0.30		1.20±0.30		1.19±0.30	
LDL(mmol/L)	2.62±0.72	2.66±0.74*		2.67±0.74		2.66±0.74		2.67±0.73		2.68±0.75	
FG(mmol/L)	5.59±1.33	5.87±1.67*		5.78±1.56		5.98±1.79△		5.85±1.68		5.96±1.65^▲^	
UA(umol/L)	357.42±77.37	351.96±77.78*		352.51±77.23		351.36±78.40		352.61±77.52		349.56±78.74	
			**Female**							
	**TN Absent** (***n*= 20880, *n*= 60.1%)**	**TN Present** (***n*= 13881, 39.9%)**	**Single TN** (***n*= 6695, 48.2%)**		**Multiple TN** (***n* = 7186, 51.8%)**		**TN <1cm** (***n*= 10480, 75.5%)**		**TN≥1cm** (***n*= 3401, 24.5%)**	
Age (years)	39.81±12.21	48.42±13.43*	46.79±12.86		49.95±13.76△		47.83±13.40		50.24±13.36^▲^	
BMI (kg/m2)	23.31±3.37	24.35±3.39 *	24.19±3.37		24.50±3.40△		24.26±3.38		24.63±3.41^▲^	
SBP (mmHg)	119.45±17.33	126.56±20.01 *	124.95±19.62		127.98±20.26△		125.92±19.85		128.35±19.85^▲^	
DBP (mmHg)	72.88±11.01	76.22±11.71 *	75.64±11.64		76.76±11.76△		75.88±11.70		77.25±11.70^▲^	
TG(mmol/L)	1.23±0.81	1.44±0.91 *	1.39±0.91		1.48±0.92△		1.42±0.93		1.47±0.88^▲^	
TC(mmol/L)	4.50±0.90	4.73±0.95 *	4.68±0.94		4.79±0.95△		4.71±0.95		4.80±0.95^▲^	
HDL(mmol/L)	1.48±0.34	1.44±0.34 *	1.44±0.34		1.44±0.35		1.44±0.34		1.43±0.34	
LDL(mmol/L)	2.52±0.73	2.69±0.78 *	2.65±0.78		2.72±0.78△		2.67±0.77		2.74±0.80^▲^	
FG(mmol/L)	5.25±0.95	5.46±1.19 *	5.42±1.13		5.50±1.25△		5.43±1.18		5.55±1.23^▲^	
UA(umol/L)	253.73±58.91	259.82±61.40 *	257.72±59.76		261.79±62.83△		259.08±60.98		262.09±62.59^▲^	

*P < 0.01 vs. Thyroid Nodule Absent group. △*P*< 0.01 vs. Single Thyroid Nodule group. ^▲^*P*< 0.01 vs. Thyroid Nodule< 1cm group.

Since the prevalence of TN differed between men and women, we further explored the metabolic characteristics by gender. Similar to the results of the total subjects, both men and women with TN tended to have worse metabolic status except for UA levels, which had an opposite trend between men and women (Table 2). Male subjects with TN showed a slightly lower UA level compared with subjects without TN (357.42±77.37 *vs* 351.96±77.78 umol/L, *p* < 0.01). Male subjects with multiple nodules and larger nodules showed an even lower UA level. Conversely, in women, UA levels were significantly higher in subjects with TN compared with subjects without TN (253.73±58.91 *vs* 259.82±61.40 umol/L, *p* < 0.01 ), and UA levels were higher in women with multiple nodules and larger nodules.

### The association of metabolic factors with occurrence of TN

**Figure 1:**
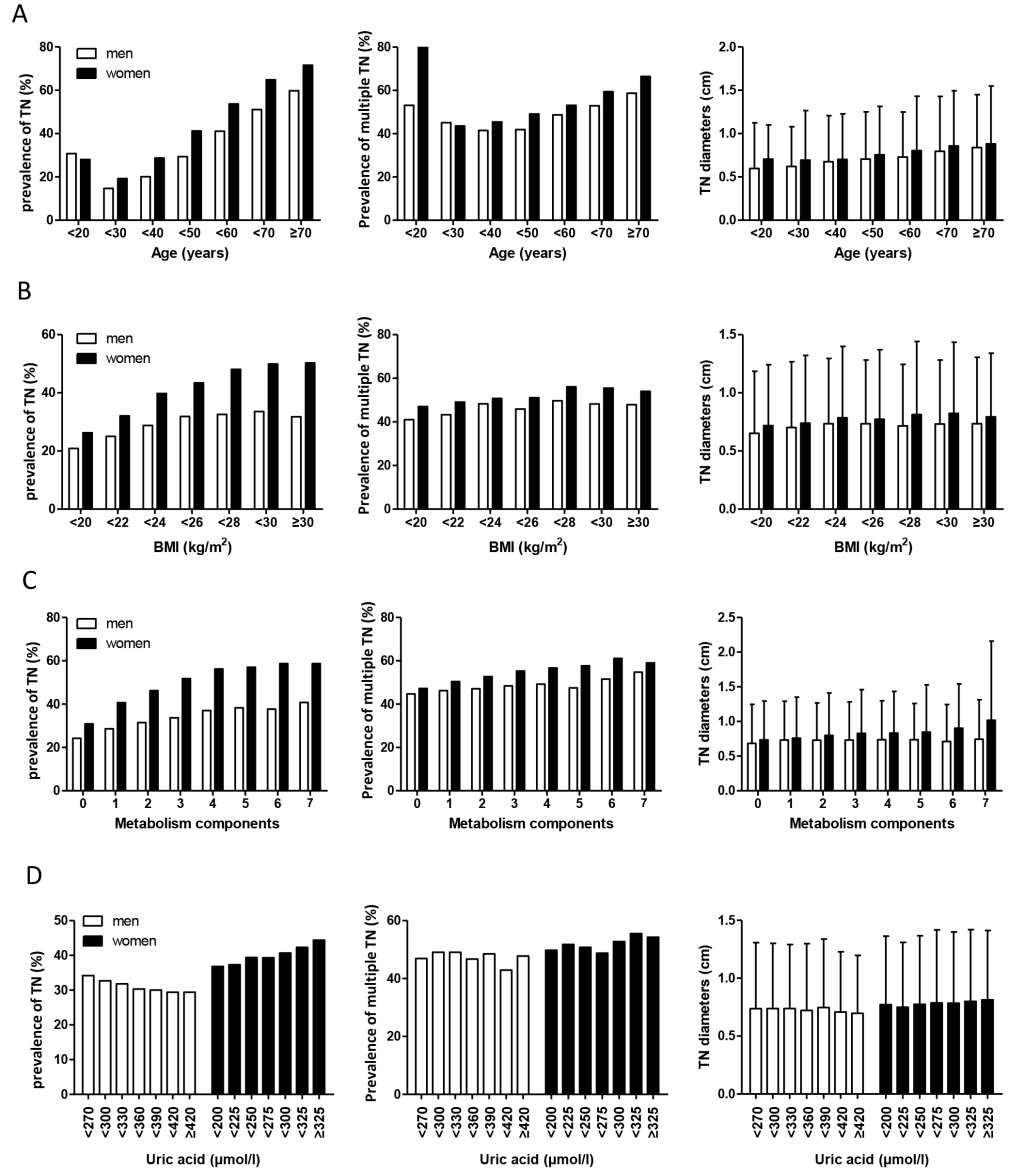
Prevalence of thyroid nodules, multiple thyroid nodules, and distribution of thyroid nodules size within the increased groups of age(**A**), BMI(**B**), metabolism components(**C**), and uric acid(**D**)

To further investigate the association between metabolic factors and occurrence of TN, we examined the occurrence of TN in groups of people with different metabolic characteristics. As shown in Figure 1, the occurrence of TN showed a U curve with the increase of age, which reached the bottom at the age range of 20-30 and sharply rise thereafter. The rate of TN in subjects aged over 70 years was about 4-fold of that in subjects aged 20-30. Among these subjects with TN, the rate of multiple TNs presented a much flatter U curve which bottomed at age 20-40, and the occurrence of TN bigger than 1cm slightly increased with age. BMI and metabolism components (including SBP, DBP, TC, TG, HDL, LDL, fasting glucose) were positively associated with a higher prevalence of TN, and women have a higher prevalence of TN than men. However, as to UA, the results were interesting. For males, prevalence of TN showed a slight decreasing trend with increasing UA. For females, prevalence of TN increased slightly with increasing UA. No significant relationships of UA with multiple TNs or large TN were observed.

### The relationship between UA and TN

**Figure 2:**
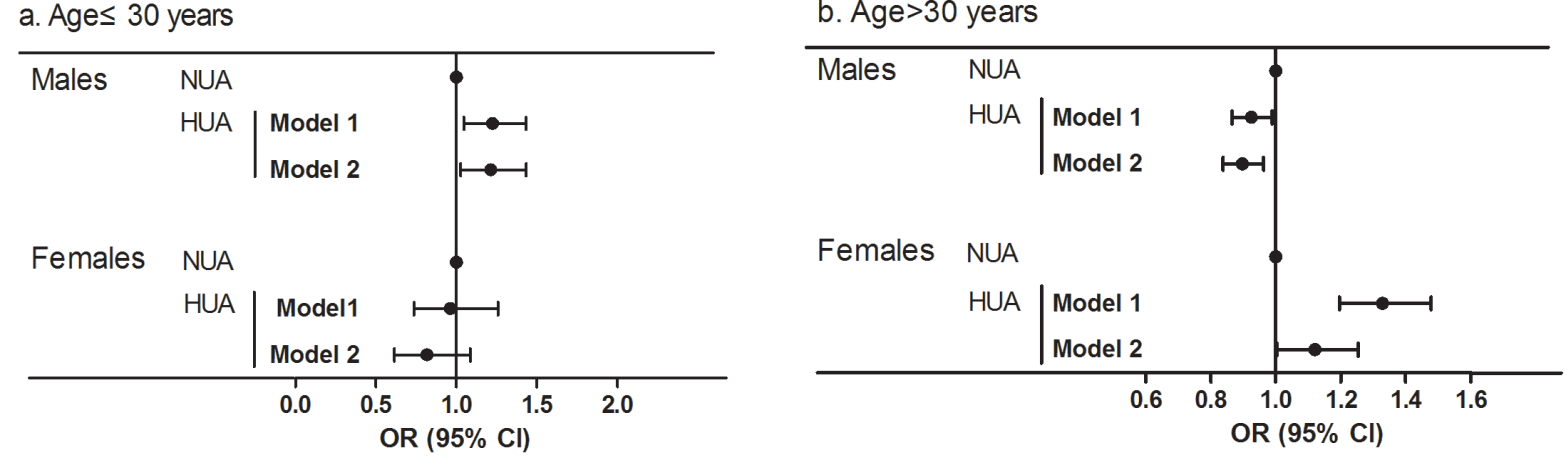
ORs and 95% CI for normal uric acid group (NUA) and high uric acid group (HUA) in the cross-sectional population stratified by sex and age (a.b) Model 1: with unadjusted value; Model 2: with adjustment for BMI, SBP, TC, TG, and FG.

To further investigate the relationship between UA and TN, we divided the subjects into two groups (age≤30 years and age>30 years) since the prevalence trend of TN was different in the two groups. The risk of TN in higher UA (HUA) group and normal UA (NUA) group was evaluated with binary and multiple logistic regression model and shown in Figure 2. In male subjects with age≤30 group, compared to NUA group, HUA group has a higher risk for TN (OR 1.225, 95CI% 1.047-1.433). The results were almost same after being adjusted for BMI, SBP, TC, TG, and FBG (AOR 1.213, 95CI% 1.027-1.432). Whereas among the female subjects younger than 30 years, HUA group showed a decrease trend in AORs when adjusted for BMI, SBP, TC, TG, and FBG though this was not significantly different (Figure 2a). However, in subjects older than 30 years, the results were totally opposite: HUA appeared to be a protective factor for TN in male subjects. The OR was 0.925 (95CI% 0.865-0.989) compared to the NUA group. After adjusting for confounding variables, AOR was 0.897, 95CI% 0.837-0.962. Among female subjects, HUA as appeared to be a risk factor for TN. Before and after adjustment AORs for the subjects in HUA group were 1.329 (95CI% 1.196-1.478) and 1.121 (95CI% 1.003-1.254) respectively.

## DISCUSSION

We analyzed the data from all people (*n* = 67,781) attending one of Health Examination Centers in Shanxi Province, China during the period from January 2012 to December 2014. We found that 30.7% of men and 39.9% of women had TN, about half of which were multi-nodularity and a quarter of which were larger than 1 cm using ultrasound examination. The rates of TN increased with age, BMI, and metabolic disorders, which was also related to the increased prevalence of multi-nodularity and larger TN. UA appeared to a protective factor for TN in men older than 30 years, but a risk factor in men younger than 30 years or in women older than 30 years.

It is reported that the prevalence of TN was estimated to be 19-67% worldwide by ultrasound, especially higher in women and old people [11–13]. A study in Hangzhou, China reported that the prevalence of TN was 34.9% in all participants, 33.9% for men and 36.9% for women. It also found that TN was related to metabolic disorders in different statistical models [14]. However, these studies have not reported the size and count of TN when reporting the prevalence of TN. Our results were consistent with other domestic and international research. Unlike other studies we further analyzed the characteristics of TN including size and count of TN. With a rapid increase of TN, the rate of thyroid cancer shows a trend of increase year by year. In 2014, approximately 63,000 new cases of thyroid cancer were diagnosed in America, which were almost twice as many as that in 2009[15]. It is predicted that papillary thyroid cancer will become the third most common cancer in women in the United States by 2019. At the same time, Chinese cancer registry annual report showed that thyroid cancer was the fourth most common cancer in women and accounted for 5.7% of all of female malignant tumors. It will create a heavy burden on the healthcare system. Since about 7-15% of TN is malignant, patients with TN should be regularly follow-up to detect thyroid cancer earlier.

Our results show associations of TN with age, BMI, or metabolic disorders. These findings are similar to previous studies. Ju-Yeon Kim et al. found that TNs increase as BMI increases, especially for female patients whose height is less than 160cm and weight over 60kg[16]. A Turkish study by Anil C et al. showed much larger thyroid and higher TN occurrence in patients with impaired glucose metabolism[17]. Although the mechanism is not very clear, the hypothesis is the internal environment disorder with insulin resistant and hyperinsulinemia, which promote thyroid cells proliferation as well as inhibit apoptosis.

In the present study, we found a relationship between UA and prevalence of TN. Serum UA is traditionally regarded as a kind of metabolic wastes and associated with metabolic syndrome whereas a recent study found that circulating UA might act as a primary antioxidant and help protect from free-radical oxidative damage[18, 19]. A marked decrease in serum UA levels would cause a decrease antioxidant capacity in serum, resulting in the damage of thyroid cells by free radicals’ oxidation and chain reaction. The occurrence of thyroid tumor may be associated with superoxide injury. A study which tested T-SOD (84.13±27.26μU/ml *vs* 102.85±33.14μU/ml, *p* < 0.05) and Mn-SOD (42.27±22.13μU/ml *vs* 65.12±28.17μU/ml, *p* < 0.01) showed that patients with thyroid tumor had a decrease in antioxidant capacity compared with healthy subjects[20]. For women, estrogen might play a role here. On one hand, estrogen can promote uric acid excretion. On the other hand, estrogen promotes proliferation and inhibits the differentiation of thyroid cells, which might become thyroid nodule cells with very low or no functions [21, 22]. In the present study, it is interesting to find that the association between TN and UA differed by gender and age groups, although the gender difference in UA and metabolic diseases or other diseases have been reported in previous studies [23, 24]. A large population-based study has shown the risk for non-alcoholic fatty liver disease (NAFLD) was higher in females than in males significantly. In a longitude study, HR was 1.249 for male and HR was 2.355 in female [25]. Another study showed that although UA was higher in men, it was only associated with severe CAD in women[26]. So, serum UA may be an antioxidant factor and affect both metabolism and endocrine system. However, the cause of gender and age differences is unknown yet; the specific mechanism needs to be further explored.

There are a number of the limitations in this study. First, all of our study population included in this analysis was from the Health Examination Center in Shanxi province of China. Some inherent bias might come from differences in residents’ living habits and diets containing iodine from other regions of China. Second, the cytological natures of thyroid nodules were unknown. According to the latest 2015 ATA management guidelines, nodules < 1 cm in greatest dimension or with low suspicion sonographic pattern or pure cystic nodules are not required for fine-needle aspiration (FNA). Consequently, most of the patients with TN were suggested to follow-up regularly instead of undergo a fine needle aspiration cytology.

In conclusion, this study expands our understanding of the thyroid nodular disease with one third residents having TN by ultrasound screening in Shanxi China. The prevalence of TN was particularly higher among people who were older, fatter, or with metabolic disorders. Serum UA appeared to be a protective factor for TN in men older than 30, but a risk factor in both men younger than 30 years and women older than 30 years. This phenomenon needs to be further investigated.

## MATERIALS AND METHODS

### Study subjects

**Figure 3:**
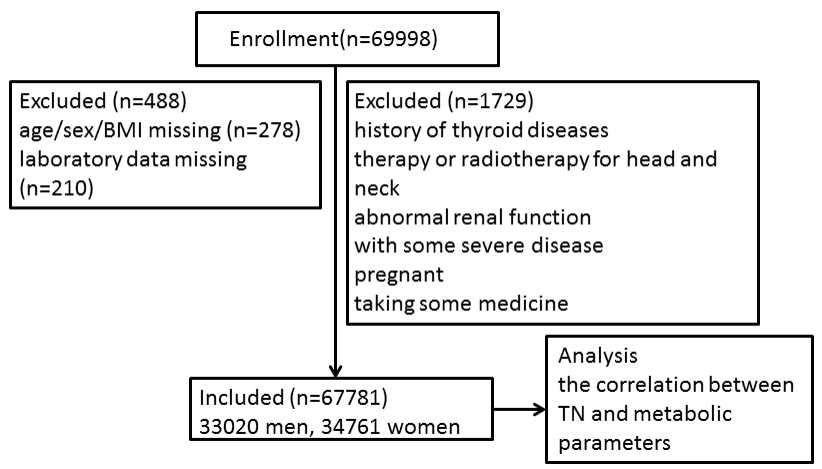
Study flow chart

In this large cross-sectional survey, samples were included from the Harmony Health Examination Center, the largest chain of health examination in Shanxi province, between January 2012 and December 2014. All of the subjects underwent a general medical examination, which included anthropometric data, thyroid ultrasound and laboratory test. The subjects came from different work places and completed a standardized health questionnaire including previous diagnoses of thyroid diseases, medication, radiotherapy and surgery therapy and other medical history. As Figure 3 showed, subjects were excluded from the study if they were: (1) with history of thyroid diseases, therapy or radiotherapy for head and neck; (2) with some severe disease such as cardiac failure, hepatic or renal dysfunction, cancer; (3) abnormal renal function that will influence the emission of uric acid; (4) pregnant or taking contraceptive or estrogens; (5) taking amiodarone, glucocorticoid, somatostatin and so on which influence thyroid function. In the end, a total of 67,781 subjects (33,020 men, 34,761 women) were included. The study protocol was developed in accordance with Helsiniki Declaration and approved by the Ethic Committee of Shanghai Tenth People's Hospital and The Second Hospital of Shanxi Medical University.

### Anthropometric indices and laboratory examination

After an overnight fast, all participants underwent a detail anthropometric evaluation including height (m), weight (kg), systolic pressure (SDP), and diastolic pressure (DBP) at 8:00 am. All of these were measured twice during the examination, and the averages of these data were used for further analysis. Body mass index (BMI) was calculated as weight/height^2^.

Venous blood samples were drawn after a fasting period of 12h. Serum triglyceride (TG), total cholesterol (TC), low-density lipoprotein cholesterol (LDL-c), high- density lipoprotein cholesterol (HDL-c), fasting glucose (FG) and uric acid (UA) levels were measured using an Olympus AU4500 automatic chemistry analyzer (Olympus Corporation, Tokyo, Japan). Definition of metabolic disorders was shown in Table 3. Thyroid ultrasound examination was performed by specific thyroid experts using a 10-MHz liner array probe (Logiq 5 Pro, GE Medical systems, USA). For each nodule, size (length, width, and depth), location, number, echogenicity, boundary, and cystic component were collected and recorded. We recorded greatest nodule dimension as TN diameter. According to these results, subjects were divided into TN present group and TN absent group. The normal UA group are subjects with UA < 420μmol/L for men and < 360μmol/L for women. Otherwise, subjects were included into the high UA group.

**Table 3:** Clinical identification of the metabolic disorders

**Metabolic components**	**Defining level**
SBP (mmHg)	>140
DBP (mmHg)	>90
TC (mg/dl)	>5.98
TG (mg/dl)	>1.21
HDL (mg/dl)	<0.9
LDL (mg/dl)	>3.12
FG (mmol/l)	>7.0

SBP, systolic blood pressure; DBP, diastolic blood pressure; TC, total cholesterol; TG, triglyceride; HDL, high- density lipoprotein cholesterol; LDL, low-density lipoprotein cholesterol; FG, fasting glucose; All of the clinical identification of the metabolic disorders were according to IDF criteria[27].

### Statistical analysis

The SPSS 20.0 software package (SPSS Inc., Chicago, IL) and GraphPad Prism 5 for Windows (GraphPad Software, San Diego, USA) were used for statistical analysis. All continuous data were expressed as mean±SD for normal distribution, and as median (interquartile range 25-75%) for skewed variables. Differences for continuous variables were assessed by independent-sample *t*-tests and category data were used by Chi square tests for percentages. Both binary and multiple logistic regression analyses were performed to assess the risk factors for TN in subjects with normal uric acid group (NUA) and high uric acid group (HUA) stratified by sex and age. Potential confounding factors including BMI, SBP, TC, TG, and FG were adjusted in the models. The unadjusted and adjusted odds ratios (ORs) and 95% confidence intervals (CIs) were calculated. All statistical tests were two-tailed, and *P* values < 0.05 were considered statistically significant.
